# Immunogenicity of Viral Vaccines in the Italian Military

**DOI:** 10.3390/biomedicines9010087

**Published:** 2021-01-17

**Authors:** Claudia Ferlito, Roberto Biselli, Vincenzo Visco, Maria Sofia Cattaruzza, Maria Rosaria Capobianchi, Concetta Castilletti, Daniele Lapa, Loredana Nicoletti, Antonella Marchi, Fabio Magurano, Anna Rita Ciccaglione, Paola Chionne, Elisabetta Madonna, Isabella Donatelli, Laura Calzoletti, Concetta Fabiani, Michela Ileen Biondo, Raffaela Teloni, Sabrina Mariotti, Gerardo Salerno, Andrea Picchianti-Diamanti, Simonetta Salemi, Sara Caporuscio, Alberto Autore, Patrizia Lulli, Francesco Borelli, Marco Lastilla, Roberto Nisini, Raffaele D’Amelio

**Affiliations:** 1 Dipartimento di Medicina Clinica e Molecolare, Sapienza Università di Roma, Via di Grottarossa 1035-1039, 00189 Roma, Italy; clau.ferlito@gmail.com (C.F.); vincenzo.visco1@uniroma1.it (V.V.); biondo_michela@yahoo.it (M.I.B.); gerardo.salerno@uniroma1.it (G.S.); andrea.picchiantidiamanti@uniroma1.it (A.P.-D.); simonettasalemi@gmail.com (S.S.); sara.caporuscio1@gmail.com (S.C.); patrizia.lulli@uniroma1.it (P.L.); raffaele.damelio@uniroma1.it (R.D.); 2 Ispettorato Generale della Sanità Militare, Stato Maggiore della Difesa, Via S. Stefano Rotondo 4, 00184 Roma, Italy; bobbiselli@libero.it; 3 Dipartimento di Sanità Pubblica e Malattie Infettive, Sapienza Università di Roma, Piazzale Aldo Moro 5, 00185 Roma, Italy; mariasofia.cattaruzza@uniroma1.it; 4 Laboratorio di Virologia, IRCCS, Istituto Nazionale Malattie Infettive “Lazzaro Spallanzani”, Via Portuense 292, 00149 Roma, Italy; maria.capobianchi@inmi.it (M.R.C.); concetta.castilletti@inmi.it (C.C.); daniele.lapa@inmi.it (D.L.); 5 Dipartimento di Malattie Infettive, Istituto Superiore di Sanità, Viale Regina Elena 299, 00161 Roma, Italy; loredana.nicoletti@iss.it (L.N.); antonella.marchi@iss.it (A.M.); fabio.magurano@iss.it (F.M.); annarita.ciccaglione@iss.it (A.R.C.); paola.chionne@iss.it (P.C.); elisabetta.madonna@iss.it (E.M.); isabella.donatelli3@gmail.com (I.D.); laura.calzoletti@iss.it (L.C.); concetta.fabiani@iss.it (C.F.); raffaela.teloni@iss.it (R.T.); sabrina.mariotti@iss.it (S.M.); 6 Centro Sperimentale di Volo, Comando Logistico, Aeronautica Militare, Aeroporto Pratica di Mare, Via Pratica di Mare 45, 00040 Pomezia, Italy; alberto.autore@gmail.com; 7 Servizio Sanitario, Reggimento Lancieri di Montebello, Esercito Italiano, Via Flaminia 826, 00191 Roma, Italy; francesco.borelli88@gmail.com; 8 Osservatorio Epidemiologico della Difesa, Ispettorato Generale della Sanità Militare, Stato Maggiore della Difesa, Via S. Stefano Rotondo 4, 00184 Roma, Italy; marco.lastilla@aeronautica.difesa.it

**Keywords:** vaccines, measles, mumps, rubella, varicella, HAV, polio, influenza, military, adults

## Abstract

Military personnel of all armed forces receive multiple vaccinations and have been doing so since long ago, but relatively few studies have investigated the possible negative or positive interference of simultaneous vaccinations. As a contribution to fill this gap, we analyzed the response to the live trivalent measles/mumps/rubella (MMR), the inactivated hepatitis A virus (HAV), the inactivated trivalent polio, and the trivalent subunits influenza vaccines in two cohorts of Italian military personnel. The first cohort was represented by 108 students from military schools and the second by 72 soldiers engaged in a nine-month mission abroad. MMR and HAV vaccines had never been administered before, whereas inactivated polio was administered to adults primed at infancy with a live trivalent oral polio vaccine. Accordingly, nearly all subjects had baseline antibodies to polio types 1 and 3, but unexpectedly, anti-measles/-mumps/-rubella antibodies were present in 82%, 82%, and 73.5% of subjects, respectively (43% for all of the antigens). Finally, anti-HAV antibodies were detectable in 14% and anti-influenza (H1/H3/B) in 18% of the study population. At mine months post-vaccination, 92% of subjects had protective antibody levels for all MMR antigens, 96% for HAV, 69% for the three influenza antigens, and 100% for polio types 1 and 3. An inverse relationship between baseline and post-vaccination antibody levels was noticed with all the vaccines. An excellent vaccine immunogenicity, a calculated long antibody persistence, and apparent lack of vaccine interference were observed.

## 1. Introduction

The military are particularly exposed to infectious diseases as a consequence of their lifestyle with close inter-individual contacts and operational activity [1]. Even if schedules vary among the armed forces of different countries, vaccination against many diseases using simultaneous inoculations of multiple antigens has been a common practice for many decades. Studies on military personnel have markedly contributed to vaccine development [2] and to global public health [3]. However, relatively few studies have faced the possible reciprocal negative or positive interference of simultaneous vaccines in adults [4]. Simultaneous inoculation of multiple antigens, including combined vaccines, is nowadays a routine practice in children, but the development of combined vaccines is generally preceded by long and accurate studies demonstrating safety and efficacy and aimed at preventing the reduction in immunogenicity as a consequence of antigens’ interference [5,6]. In fact, studies in children receiving multiple vaccines demonstrated the possibility of reduced antibody response caused by antigen interference [7,8,9]. A negative interference of tetanus/diphtheria and pneumococcal CRM197-conjugate vaccine was suggested [10]. On the contrary, a possible positive interference has also been recently reported in the US military [4], suggesting that the study of adults undergoing multiple, simultaneous vaccinations represents a valuable model to test this issue.

In the 1990s, the epidemiological situation of infectious diseases in the Italian military population was characterized by the net increase in varicella, rubella, and measles, as documented by the comparison of the period 1991–1995 with the period 1976–1980 [11]. On this basis, in 1998, the military health authorities decided to introduce the trivalent live measles/mumps/rubella (MMR) vaccination, later associated to varicella, in the compulsory schedule for all recruits, irrespective of the possible already established protection for natural immunization [12]. The effectiveness of the trivalent MMR was promptly demonstrated by observing 95% of measles and rubella and 70% of mumps cases’ reduction as early as two years after the introduction of MMR [13]. Moreover, even the mumps vaccine’s effectiveness would have probably been higher if in the effectiveness calculation, vaccine-induced mumps-like clinical cases caused by the not sufficiently attenuated mumps vaccine strain Urabe Am9, included in the vaccine used at that time, had not been considered [14]. Although in the pivotal study by Amanna et al. [15], the very long duration of antibodies induced by viral antigens was clearly calculated, and in addition, pre-licensure studies have indicated the persistence of antibodies induced by the MMR vaccine as lifelong, the matter has been poorly studied [16]. Even the possible interference by other concomitantly administered vaccines has only rarely been investigated.

A progressive lowering of hepatitis A virus (HAV) circulation was observed in Italy and documented by studies on military population at approximately 10-year intervals [17]. However, the HAV vaccination was added to the compulsory vaccine schedule for the Italian military in 1998 [12] considering that HAV is the most frequent vaccine-preventable infection in travelers [18] and military personnel operate in many international scenarios. Although the vaccine’s immunogenicity is high and the anti-HAV antibody persistence is generally considered long-lasting, the possible negative or positive interference exerted by other viral or bacterial vaccines has not yet been thoroughly investigated.

Up until the end of the last century, the polio vaccine used for Italian infants was the live trivalent Sabin’s vaccine (oral polio vaccine (OPV)), then replaced in 2003 by the Salk’s trivalent inactivated polio vaccine (IPV) [19]. In the military, a booster of IPV was introduced in 1998 in the compulsory vaccine schedule of permanent staff for possible deployment abroad [12]. The efficacy of the polio vaccine has been so widely demonstrated that the World Health Organization (WHO) has already certified the global eradication of polioviruses types 2 [20] and 3 [21]. The wild type 1 is still present only in Pakistan and Afghanistan, even though the number of cases in 2019 increased fourfold compared to 2018 [22]. However, to our knowledge, the immunogenicity of an IPV booster administered to adults primed at infancy by OPV and the persistence of the induced antibodies have not yet been explored or calculated, respectively.

Finally, influenza vaccination is offered on a voluntary basis, mainly to Italian military personnel deployed abroad. However, although influenza control is considered a crucial issue by the military [23] and civil–military collaboration on influenza dates back to at least the Second World War [24], only a limited number of world countries have included influenza vaccination in the compulsory vaccination program of their military personnel [25]. Nevertheless, the study of the immune response to this vaccination in the military allows to gain more insights on a vaccination model which is unique. In fact, the immunization is repeated yearly due to the annual variability of viral strains.

The aim of the current study, which is part of a larger survey on the safety [26] and immunogenicity [27,28] of vaccinations in the Italian military, was therefore to analyze immunogenicity and evaluate whether the current vaccination schedule for the military is adequate and the possible reciprocal positive and negative interferences of inactivated and living viral vaccines in two groups of Italian military personnel. The first group was represented by students from military schools working in Italy, and the second group was composed of soldiers who worked abroad (Lebanon) for nine months and received additional vaccinations before departure.

## 2. Materials and Methods

### 2.1. Study Population

From September 2012 to June 2014, two groups of Italian military personnel were enrolled. The first group was represented by 108 newly recruited students from military schools, residing in Italy for at least 3 years, and the other was formed by 72 soldiers deployed abroad (Lebanon) for nine months.

Blood samples were collected after obtaining informed consent pre-vaccination (T0), at 1 month (T1) and 9 months (T2) post-vaccination in group 1, whereas they were collected at T0 and T2, upon returning from the international mission in Lebanon, in group 2.

All subjects gave their informed consent for inclusion before they participated in the study. The study was conducted in accordance with the Declaration of Helsinki, and the protocol was approved by the Ethics Committee of the Italian Ministry of Defense in July 2011 and registered on clinicaltrials.gov in 2012 with the identifier NCT01807780.

### 2.2. Vaccination Schedule

At enrollment, informed consent and medical history of all individuals were collected. The vaccination schedule was personalized based on information on the history of infectious diseases and already-received vaccinations; moreover, the vaccination schedule was even tailored based on the type of employment, either national or international. Administered vaccines were the following: tetanus/diphtheria (Td, DifTetAll-Novartis Vaccines and Diagnostics, Siena, Italy), inactivated polio (Imovax polio-Sanofi Pasteur MSD SpA, Roma, Italy), measles/mumps/rubella (MMR, Priorix-GlaxoSmithKline SpA, Verona, Italy), chickenpox (Varivax-Sanofi Pasteur MSD SpA, Roma, Italy), polysaccharide tetravalent (A, C, W_135_, Y) meningococcal meningitis (Mencevax-Pfizer Srl, Latina, Italy), hepatitis A (HA, Epaxal-Crucell Italy Srl, Baranzate, Italy), hepatitis B (HB, Engerix B-GlaxoSmithKline SpA, Verona, Italy), influenza (Fluad-Seqirus Srl, Siena, Italy), and typhoid (Vivotif Berna-PaxVax Ltd., Birmingham, UK). Vaccines were generally administered on the same day (in different arms), but in a few cases, up to two weeks apart. However, a considerably lower number of subjects than expected were immunized with the vaccines (Table 1) because of previous vaccination records and history of infectious diseases.

### 2.3. Measles/Mumps/Rubella Antibody Analysis

For measles/mumps/rubella antibody determination, commercial enzyme-linked immunosorbent assay (ELISA) kits were used: Enzygnost^®^ Anti-Measles Virus/IgG (Siemens Healthcare Diagnostics GmbH, Marburg, Germany); Enzygnost^®^ Anti-Mumps Virus/IgG (Siemens Healthcare Diagnostics GmbH, Marburg, Germany); Enzygnost^®^ Anti-Rubella Virus /IgG (Siemens Healthcare Diagnostics GmbH, Marburg, Germany). Despite the fact that a correlate of protection for mumps has not yet been found, and for measles and rubella, has only been identified in the microneutralization assay, the putative quantitative threshold for seropositivity considered here was ≥0.15 IU/mL for measles, a titer of ≥1:230 for mumps, and ≥4 IU/mL for rubella [29]. However, considering that antibody levels were defined as low positive (equivocal) at 0.15–0.35 IU/mL for measles, a titer of 1:230 to 1:500 for mumps, and 4–7 IU/mL for rubella, according to Davidkin et al. [29], the more restrictive limits of ≥0.35 IU/mL, 1:500, and ≥7 IU/mL, for measles, mumps, and rubella, respectively, were actually adopted as the putative thresholds for protection (Table 2). Responders were defined the subjects able to at least quadruplicate post-vaccination antibody levels [30], irrespective of the baseline antibody levels.

### 2.4. Varicella Antibody Analysis

For varicella antibody determination, a commercial ELISA kit was used: Enzygnost^®^ Anti-VZV/IgG (Siemens Healthcare Diagnostics GmbH, Marburg, Germany); Results of ≤5 IU/mL were interpreted as negative [31] (Table 2).

### 2.5. HAV Antibody Analysis

For HAV antibody determination, a commercial ELISA kit was used: Enzygnost^®^ Anti-HAV (Siemens Healthcare Diagnostics GmbH, Marburg, Germany). The quantitative cut-off value for seropositivity was ≥0.01 IU/mL [31] (Table 2).

### 2.6. Polio Antibody Analysis

#### 2.6.1. Cells and Viruses

Vero E6 cells (European Collection of Cell Cultures, Salisbury, UK) were maintained in Modified Eagle Medium (MEM) supplemented with 10% heat inactivated fetal calf serum (FCS) (Gibco, Thermo-Fisher Scientific, Waltham, MA, USA) at 37 °C in a humidified atmosphere. In our assays, the two polioviruses Sabin serotypes 1 and 3 were used (kindly provided by Dr Giovanni Rezza from the Istituto Superiore di Sanità, Rome, Italy). The virus stocks were propagated in Vero E6 cells and were harvested at 48 h post-infection, when 70–80% of cell monolayers had been killed. After freezing and thawing three times, cell lysates were clarified by low-speed centrifugation, aliquoted, and stored at −70 °C. The poliovirus Sabin type 1 virus stock used for all the experiments at titered 10^7.8^ 50% tissue culture infectious doses per milliliter (TCID_50_/mL). The poliovirus Sabin type 3 virus stock used for all the experiments at titered 10^6.5^ TCID_50_/mL.

#### 2.6.2. Microneutralization Assay

The presence of poliovirus-neutralizing antibodies was assessed according to WHO guidelines [32,33]. Briefly, all serum samples, stored at −20 °C until use, were heat-inactivated (56 °C for 30 min) and serially twofold diluted from 1:4 to 1:4096 in MEM containing 2% heat-inactivated FCS (final volume: 50 μL) in 96-well plates. Each well dilution was challenged with 50 μL of type 1 or type 3 poliovirus Sabin strain (100 TCID_50_ per well, 1 plate for each serotype of poliovirus) and incubated at 37 °C for 2 h in a CO_2_ incubator. After this first incubation period, each serum/viruses mix was transferred onto 24-h-old Vero E6 cells in 96-well plates and incubated in a humidified CO_2_ incubator at 36 °C for 5 days.

An in-house reference serum was included in each test run to control the reproducibility of results, a 1:4 dilution of each serum without virus was included for each serum tested to check serum toxicity, and challenging viruses were back-titrated in each test run to control the TCID_50_ dose.

The neutralizing antibody titer of the serum against each type of poliovirus was determined as the endpoint dilution of the serum that inhibited the poliovirus infection, observed by cytopathic effect of inoculated cells. The neutralizing antibody assay is the method of choice to determine the immune status against poliovirus. Although the precise antibody titer that is necessary for protection is unknown, it is accepted that 1:4–1:8 of type-specific neutralization of an infection in a cell culture is putatively protective [31]; however, in the current study, the more stringent cut-off of 1:8 was chosen [34] (Table 2). Responders were defined as the subjects least able to at quadruplicate baseline antibody titer at T2 [35].

### 2.7. Influenza Antibody Analysis

Sera were analyzed using the hemagglutination inhibition (HAI) test, according to standard procedures [36]. Briefly, sera were pre-treated with receptor-destroying enzymes in order to remove the non-specific agglutinins and heated at 56 °C for 30 min. Antigens used for testing were the same or antigenically equivalent to those included in the vaccine formulation (H1/A/California/7/09, H3/A/Texas/50/12, and B/Massachusetts/2/12). Each serum was tested at a standard dilution of 1:10; the assay was carried out in a U-shaped 96-well microtiter plate, with 4 units of virus and human red blood cells at a concentration of 0.75%, incubated at room temperature for 60 min following red blood cell addition.

Geometric mean HAI titers, seroprotection rate (the percentage of vaccine recipients with a serum HAI titer of at least 1:40 after vaccination), seroconversion rate (the percentage of vaccine recipients with a post-vaccination increase in HAI titers by at least a factor of 4 as compared with pre-vaccination titers), and seroconversion factor (the post-vaccination antibody titer divided by the pre-vaccination antibody titer) were calculated. A seroprotection rate exceeding 70%, a seroconversion rate exceeding 40%, and a seroconversion factor exceeding 2.5 were considered as cut-off levels of immunogenicity for 18–60-year-old adults, according to the guidelines of the European Committee for Proprietary Medicinal Products (CPMP) for the assessment of influenza vaccines. To meet the CPMP guidelines, each of the vaccine antigens must meet at least one of the above criteria [37].

### 2.8. HLA Typing

DNA extraction from ethylenediaminetetraacetic acid (EDTA)-treated whole blood was performed using a Qiagen kit (Qiagen Inc., Germantown, MD, USA) as per the manufacturer’s protocol. *HLA-A, -B,* and *-C* loci, were genotyped at low molecular resolution (at allele group level) using polymerase chain reaction–sequence-specific oligonucleotide (PCR-SSO) assays, based on the reverse hybridization principle (typing kits INNO-LiPA^®^ *HLA-A, -B, -C,* Fujirebio Europe N.V.). *HLA-DRB1* and *-DQB1* loci were typed using the PCR–sequence-specific primers (PCR-SSP) method (micro SSP DNA typing Kits One Lambda Inc., Canoga Park, CA, USA).

HLA allele frequencies were estimated by direct counting in the study subjects. The alleles were identified assuming that *HLA-A, -B, -C, DRB1* and *DQB1* loci have no blanks. When a single allele was found, the individual was considered homozygous for that allele.

### 2.9. Statistical Analysis

Geometric mean concentrations (GMCs) and their 95% confidence intervals were calculated to describe the IgG ELISA response for each group, pre- and post-vaccination. Antibody concentration below the ELISA limit of detection was assigned a titer of 0.001 μg/mL for analyses. All comparisons between groups were made with log-transformed data, using Student’s *t*-test. Categorical variables were analyzed by Yates-corrected, two-tailed Pearson’s χ^2^ test. Correlation was explored using the linear regression test. *p* values ≤ 0.05 were considered significant. Statistical analysis was performed using the program GraphPad Prism package 5.0 version (GraphPad Software Inc. San Diego, CA, USA). Multivariate analysis was performed using the parameter responders/non-responders as the dependent variable and as covariates the other variables. The software used for the test was R: Copyright 2004–2013, The R Foundation for Statistical Computing. The half-life of vaccination-induced antibodies was calculated with the following equation: log (antibody concentration) = α + β × years + ε, where α represents the mean log concentration at the time of vaccination; β represents decay rate, and ε represents the error term (β = [log (antibody concentration) − α − ε]/years [38].

## 3. Results

### 3.1. Study Population

Group 1 consisted of 108 subjects recruited from military schools (mean age ± standard deviation 21.05 ± 2.5; males, 96 (89%)) and group 2 of 72 members of the Regiment “Lancieri di Montebello” (located in Rome), actively engaged in a nine-month-long international mission in Lebanon (mean age ± standard deviation 31.2 ± 4.6; males, 69 (96%)). Mean age was significantly different between the two groups (*p* < 0.0001), while male/female rate was not.

The number of administered vaccines is reported in Table 1 and the number of co-administered vaccines in both groups was as follows: two (1.85%) subjects in group 1 received only one vaccine; 33 (30.6%) subjects in group 1 and 11 (15.3%) subjects in group 2 received two vaccines; 55 (50.9%) subjects in group 1 and 43 (59.7%) subjects in group 2 received three vaccines; 16 (14.8%) subjects in group 1 and 14 (19.4%) subjects in group 2 received four vaccines; whereas two (1.85%) subjects in group 1 and four (5.5%) subjects in group 2 received five vaccines.

Based on blood samples’ availability at the three times (T0, T1, and T2), 49 subjects for MMR vaccine, 8 for varicella, and 56 for HAV were studied in group 1; the response to polio types 1 and 3 was explored in 52 subjects, equally distributed between the two groups, and the response to influenza was studied in 72 subjects, all belonging to group 2.

### 3.2. MMR

**Figure 1:**
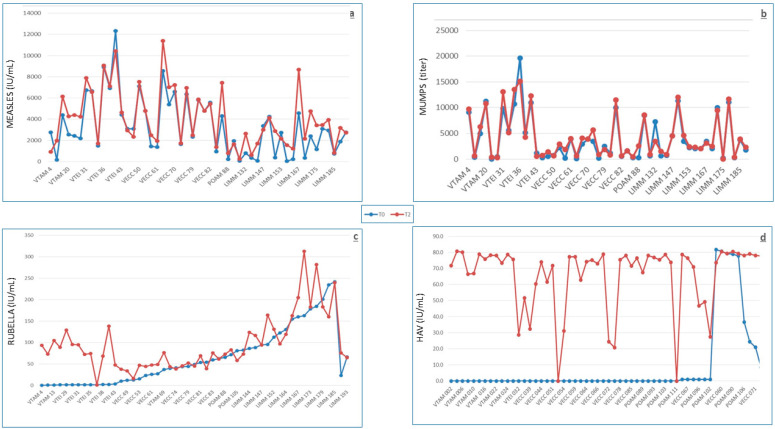
Antibody levels at T0 (before vaccination, blue points/lines) and at T2 (9 months after vaccination, red points/lines) against HAV (**a**), measles (**b**), mumps (**c**), and rubella (**d**).

At baseline, 40/49 (82%) subjects already had positive measles antibody levels (≥0.35 IU/mL), whereas at T1 and T2, they were 47/49 (96%) (*p* < 0.02, T0 vs T1 and T2). These individuals were considered putatively protected (Table 2). The antibody levels at T0 and T2 are reported in Figure 1a. The geometric mean concentrations (GMCs) at T0, T1, and T2 were 1.736, 2.826, and 2.283 IU/mL, respectively. There were eight responder subjects (subjects showing at least fourfold post-vaccination antibody increase)—seven at T1 (14%) and six at T2 (12%). Despite the fact that none of the subjects with putatively protective baseline antibody levels could quadruplicate antibody levels at T2, 5/9 subjects with low/lacking baseline antibody levels could (*p* < 0.00002). In 13/40 (32.5%) subjects with baseline putative protective antibody values, significantly (*p* = 0.03) reduced antibody levels were observed at T2. The baseline GMCs of the 44 subjects who could not quadruplicate, at T2, the post-vaccine anti-measles antibody value were over 23-fold higher than the GMCs of the five subjects who could (*p* < 0.0001) (Table 3).

At baseline, 40/49 (82%) subjects already had positive mumps antibody titers (≥1:500), whereas at T1 and T2, they were 48/49 (98%) (*p* < 0.02, T0 vs T1 and T2). These individuals were considered putatively protected (Table 2). The antibody levels at T0 and T2 are reported in Figure 1b. The geometric mean titers (GMTs) at T0, T1, and T2 were 1:1868, 1:4427, and 1:3166, respectively. Responder subjects at T1 were 11/49 (22%, including two subjects with baseline antibody levels > 1:500 and all the nine subjects with baseline antibody levels < 1:500); however, only 7/49 (14%) remained at T2. None of the subjects with baseline antibody levels > 1:500 could quadruplicate antibody levels at T2, whereas 7/9 subjects with low/lacking baseline antibody levels could (*p* < 0.0000001). The baseline GMTs of the 42 subjects who could not quadruplicate the post-vaccine anti-mumps antibody value at T2 were over 23-fold higher than the GMTs of the seven subjects who could (*p* < 0.0001) (Table 3).

At baseline, 13/49 (26.5%) subjects had negative rubella antibody levels, whereas the remaining 36 were putatively protected (>7 IU/mL), and at T1 and T2, all individuals but one (98%) resulted to be putatively protected (*p* < 0.002) (Table 2). The antibody levels at T0 and T2 are reported in Figure 1c. The GMCs at T0, T1, and T2 were 23, 72, and 76 IU/mL, respectively. Responder subjects at T1 counted at 16/49 (33%, including 12/13 subjects with baseline negative antibody levels and 4/36 subjects with baseline antibody levels >7 IU/mL), whereas only 12/49 (24.5%) remained at T2, all with negative baseline antibodies. None of the 36 subjects with putative protective baseline antibody levels could quadruplicate antibody levels at T2, whereas 12/13 subjects with baseline antibody levels <7 IU/mL could (*p* < 0.0000001). In 9/36 (25%) subjects with baseline putative protective antibody values, significantly (*p* = 0.0015) reduced antibody levels were observed at T2. The baseline GMCs of the 37 subjects who could not quadruplicate the post-vaccine anti-rubella antibody value at T2 were over 45-fold higher than the GMCs of the 12 subjects who could (*p* < 0.0001) (Table 3).

At baseline, 21/49 (43%) subjects already had putative protective antibody levels for all three viral antigens, whereas at T2, they were 45/49 (92%). The response at T2 of the baseline putatively protected subjects was lower than that of the subjects without baseline putative protective levels, but the difference was not significant. Due to the low number of subjects, no logistic regression could be done.

No post-vaccine serious clinical adverse events (only one local reaction [26]) nor cases of measles, mumps, or rubella have been described.

### 3.3. Varicella

At baseline, 6/8 (75%) subjects already had putative protective antibody levels (≥5 IU/mL), whereas they were 8/8 (100%) at T1 and T2 (Table 2, *p* = NS). The GMCs at T0, T1, and T2 were 9.11, 27.7, and 30.98 IU/mL, respectively. No post-vaccine clinical adverse events [26] nor cases of varicella have been described.

### 3.4. HAV

At baseline, 8/56 (14%) subjects already had putative protective antibody levels (≥0.01 IU/mL, Table 2), whereas putatively protected subjects increased to 55/56 (98%) and 54/56 (96%) at T1 and T2 (*p* < 0.0000001 for both, T0 vs T1 and T0 vs T2), respectively. The antibody levels at T0 and T2 are reported in Figure 1d.

The geometric mean concentrations (GMCs) at T0, T1, and T2 were 0.00034, 0.063, and 0.052, respectively. Ten subjects were non-responders, including all the individuals with baseline antibodies at putative protective level (*p* < 0.0000001, baseline putatively protected vs unprotected individuals). Moreover, the subjects with higher baseline GMCs had significantly lower responses at T2 than the subjects with lower baseline antibody levels (*p* = 0.0345; Table 3). The logistic regression, carried out by using the parameter responders/non-responders as the dependent variable and three or more concomitant vaccinations and the titers at T0 as covariates, showed that the probability of response decreases with the increase in baseline antibody titers (odds ratio (OR) 0.74, 95% CI 0.55–0.98, *p* = 0.0381).

No post-vaccine clinical adverse events [26] nor cases of HAV infection have been observed.

### 3.5. IPV

Anti-polio antibodies types 1 and 3 were analyzed in 52 military individuals, equally distributed between the two groups. Seven subjects out of group 2 (26 subjects) did not receive the recent polio booster, but they were also considered in order to check for possible anti-polio immune modulation by vaccines other than polio. Moreover, the timing of the polio booster in 12 subjects was not comparable to that of the remaining seven and that of the first group’s subjects (who were inter se similar). Thus, the inter-group comparison was only performed on 25 subjects of group 1 and six subjects of group 2, who had the same vaccine history and for whom the samples at T2 were available.

**Figure 2:**
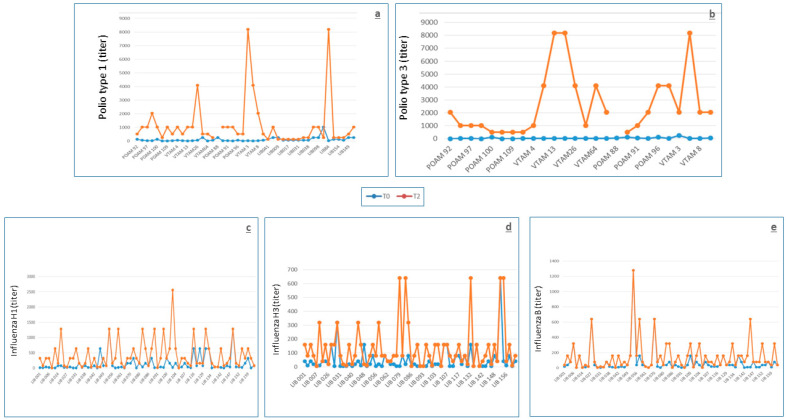
Antibody levels at T0 (before vaccination, blue points/lines) and at T2 (9 months after vaccination, orange points/lines) against polio type 1 (**a**) and polio type 3 (**b**), Influenza H1 (**c**), Influenza H3 (**d**), and Influenza B (**e**).

All the subjects except four from group 1 had baseline titers ≥ 1:8 for anti-type 3 antibodies (the antibody titer considered here as the threshold for putative protection, Table 2) at T1 and T2. All the subjects of the first group showed at least a fourfold post-vaccine antibody increase (a criterion to define immune response) towards both poliovirus types 1 and 3 at T1 as were all but one (who was unable to quadruplicate the antibody titer towards type 1) at T2, whereas the response at T2 of the second group was lower; in fact, all six subjects could at least quadruplicate antibody titer to type 1, but only 2/6 to type 3. Moreover, even for the response to type 1, those who could only quadruplicate were 4/6 (66%) and 3/26 (11.5%) for groups 2 and 1, respectively (*p* = 0.01655). The antibody levels at T0 and T2 are reported in Figure 2a,b for type 1 and type 3, respectively. Out of the seven subjects who had not received the recent polio booster, six maintained the same baseline titer at T2 and one showed a one-dilution reduction.

At T0, the six subjects of the second group had an anti-type 1 and 3 mean antibody level significantly higher than the 25 subjects of the first group, whereas the opposite was observed at T2. In fact, the GMTs towards polio type 1 at baseline were 36 and 90.5 and the GMTs towards polio type 3 were 23 and 128 for groups 1 and 2, respectively (*p* < 0.0001 group 1 vs group 2), whereas the anti-type 1 T2/T0 antibody ratio was 52 and 5 and the anti-type 3 T2/T0 antibody ratio was 184 and 3.7 for groups 1 and 2, respectively (*p* < 0.0001 group 1 vs group 2) (Table 4). Considering the response at T2, there were only 5/31 (16%) non-responder subjects (4/6 of group 2 in the response towards type 3 (*p* = 0.0002244 group 1 vs group 2 in the response to type 3) and only one of group 1 in the response towards type 1 polio virus (*p* = NS group 1 vs group 2 in the response to type 1). Due to the relatively low number of analyzed subjects, no logistic regression could be done.

No post-vaccine adverse events were observed [26].

### 3.6. Influenza Vaccine

The antibody response to the trivalent subunit MF59-adjuvanted influenza vaccine was adequate, fulfilling all the parameters of seroprotection rate, seroconversion rate, and seroconversion factor according to CPMP (Table 5). The antibody levels at T0 and T2 are reported in Figure 2c–e for H1, H3, and B, respectively.

By assuming the at least fourfold increase in the seroconversion rate as an equivalent of immune response, we could observe 16 non-responders to all (H1, H3, and B) vaccine antigens, 16 responders to all antigens, 21 non-responders towards 2/3 antigens, and 19 non-responders to 1/3 antigens. The baseline GMTs of the 35, 30, and 44 subjects who could not quadruplicate, at T2, the post-vaccine antibody titer towards H1, H3, and B, respectively, were 5.6-, 3.5-, and 2.2-fold higher, respectively, than the GMTs of the subjects who could (*p* < 0.0001; *p* = 0.0005; *p* = 0.012) (Table 4).

The logistic regression, carried out by using the parameter responders/non-responders as the dependent variable and age, number of concurrent vaccinations, and the titers at T0 as covariates, showed that the probability of response decreases with the increase in baseline antibody titers (*p* = 0.0020 for H1; *p* = 0.0024 for H3; *p* = 0.0261 for B). Moreover, the response to antigen B decreases with increase in age (*p* = 0.0074) (Table 6). Thirteen (18%) subjects had baseline antibodies towards the three antigens (H1, H3, and B) at putative protective levels (≥1:40) [39], whereas there were 50 (69%) at T2.

Despite the influenza vaccine being the most reactogenic vaccine (details have been reported elsewhere [26]), no serious post-vaccine clinical adverse events nor cases of influenza-like illness (ILI) have been described.

### 3.7. Calculated Antibody Half-Lives

The calculated half-lives of vaccine-induced antibodies were 690 (95% CI 225–1380) years for HAV, 92 (95% CI 62–180), 111 (95% CI 50–230), and 81 (95% CI 58–190) years for measles, mumps, and rubella, respectively, and 34.5 (95% CI 15–60) and 57.5 (95% CI 24–90) for polio types 1 and 3, respectively. It must be underlined that these data arise from 56 subjects for HAV and 49 for measles, mumps, and rubella but only 26 subjects of group 1 for polio, for whom the samples collected before and 1 month and 9 months after the booster were available. No antibody half-life calculation has been performed for varicella, considering the low number of vaccinated subjects, nor for influenza, considering that it is a yearly repeated vaccination.

### 3.8. Association of HLA with Antibody Response

No association between HLA antigens and immune response was found for any viral vaccine explored.

## 4. Discussion

The analysis of antibody levels against MMR in 49 young Italian subjects showed a high rate of subjects with putative protective levels before vaccination. In fact, 82%, 82%, and 73.5% of the subjects already had antibodies above the putative threshold for protection against measles, mumps, and rubella, respectively, and 43% had protective antibody levels for all three viral antigens. Considering that the MMR vaccination was introduced for infants in Italy in 1999 [40] and that the subjects described here had been vaccinated in 2013 at a mean age of over 21 years old, it can be argued that the large majority had antibody levels as a consequence of natural immunization. In fact, the circulation of measles and rubella viruses in Italy at the beginning of this century was high, and the relative infections were still very frequent, with an observed shift towards higher ages, albeit not always clinically evident [41]. The long antibody persistence that we estimated on the basis of the calculated half-lives of 62, 50, and 58 years for measles, mumps, and rubella, respectively, is in line with the observations of Amanna et al. [15]. Even if our estimates are markedly lower than those previously reported [15], the expected lifelong persistence of these antibodies strongly indicates that booster doses might not be required for this vaccination, at least in healthy people. This suggestion is not in line with some reports showing a drop in circulating antibodies [16,42,43]. Interestingly, in this line, there was an observation that a progressive decrease in antibody titers is not associated to a significant reduction in neutralizing antibodies when neutralizing antibodies were measured in parallel with specific antibodies by ELISA [44]. The uselessness of booster doses is also suggested by the observation that the effect of a third booster is transient and is not required to maintain specific antibodies above the threshold of putative protection for a longer time [44]. Long-lasting specific antibodies may also be involved in the rareness of post-vaccine adverse events. In addition to the general safety of the MMR vaccine, preexisting antibodies at putative protective titers observed in many subjects probably act as live vaccine-inactivating agents, reducing their capacity to interact with target cells. However, some doubts arise when considering the inverse relationship between baseline antibody levels and immune response and, even more so, the significant reduction in antibody levels in one third and one fourth of the subjects with putative protective antibody levels at baseline following measles and rubella vaccinations, respectively. Such doubts might derive from a vaccination strategy indiscriminately applied and not based on previous antibody level screening, which is a procedure that has also been considered for cost effectiveness reasons [45]. This study was designed without control groups receiving single vaccinations, which would have enabled the analysis of vaccine reciprocal interference; however, the excellent immune response observed allows to infer a lack of negative interference by simultaneously administered vaccines, as already reported in the literature [46].

The HAV baseline seropositivity, generally induced by natural immunization as the vaccination is not included in the Italian infant vaccination schedule, showed a net increase when compared with the military population analyzed over 10 years before [17], being almost threefold higher (14% vs 5.3%). It is difficult to identify the possible reason(s) for such an increase considering that in Italy, a progressively decreasing epidemiological trend for HAV has been observed in recent decades and following the introduction, in 2012, of the HAV vaccine. In the Italian general population, the rate of HAV infection in 2012 was 0.8 per 100,000 [47]. According to the Italian surveillance system for hepatitis, the main risk factors for HAV infection have been traditionally represented by consumption of raw shellfish, travel to endemic areas, and male homosexuality [48]. Since 2013–2014, a new risk factor, represented by the consumption of contaminated mixed frozen berries, appeared and was responsible for a large outbreak in different Italian regions and European countries [49]. The first cause of increased occurrence may be represented by the high prevalence of military personnel (nearly 50% of those vaccinated for HAV) coming from Campania and Puglia, the two Italian regions where the habit of raw seafood consumption is still traditionally present [50]. However, the distribution of these individuals is not significantly different between the two groups, one with positive and the other with negative baseline antibody levels. As a consequence, this cause was ruled out, as was the consumption of contaminated frozen berries, considering that these subjects were studied one year before the outbreak. Other foods which may be contaminated and transmit HAV infection are poorly washed fresh vegetables, grilled meat, and fresh cheese [51], as recently thoroughly discussed in relation to the description of a family cluster of HAV infections, not associated with raw shellfish or berries [52]. Nonetheless, in the Italian military, detailed information on these issues as well as on the prevalence of male homosexuality is lacking. The vaccine’s immunogenicity has been confirmed to be very high, as already described [53]. No post-vaccine case of HAV infection in the Italian military has been described, despite the military representing professional travelers and HAV being recognized as the biggest threat for travelers [19], thus allowing to confirm a very high vaccine-induced protection. A significantly better response was observed in subjects with low rather than high baseline antibody levels, as already observed for quadrivalent meningococcal polysaccharide [27] and for tetanus/diphtheria [28] vaccines. The calculated half-life of antibodies is in line with the direct measurement of antibody levels at 22 years post-vaccination [54], which has shown a very high percentage of still-protected individuals, thus suggesting that a booster would be unnecessary, considering the protection induced by immune memory [55]. Lack of interference by concurrent vaccinations may be inferred from the results of the logistic regression.

In this study, volunteers were vaccinated with the trivalent inactivated polio vaccine, but only the anti-type 1 and 3 antibodies were measured because type 2 has been officially considered eradicated by the WHO, which recommended the global withdrawal of the type 2 component of OPV in April 2016 [20]. We could measure anti-type 3 antibodies, last isolated in 2012, since type 3 was declared eradicated by the WHO in October 2019 [21]. Anti-polio antibodies types 1 and 3 were present in all tested individuals, except from only four individuals of group 1 who lacked protection against poliovirus type 3. These individuals showed specific antibodies at a titer of 1:4, which is considered just as protective [31]. Considering that in Italy, the compulsory polio vaccination for infants was modified from OPV to a transient regime of OPV/IPV and then IPV only at the end of the last century [19], we may argue that the baseline high level of protection could be a consequence of priming immunization at infancy with four doses of Sabin’s OPV, which was present in the Italian infant schedule until 1998. The level of baseline protection observed here is markedly higher than that found in an Italian population whose blood was collected in 2009 [19], approximately in the same time period of the current study. In fact, by comparing the 26 subjects of group 1 of the current study, for whom the vaccination history is comparable to that of the 99 subjects, age range 15–43 years, of the cited study (only four doses of OPV at infancy), the prevalence of putatively protected individuals (neutralizing antibody titer ≥ 1:8) was 100% vs 78.8% for type 1 and 84.6% vs 38.4% for type 3, respectively (*p* = 0.02292 for type 1; *p* = 0.00006931 for type 3). The reasons for such discrepancy are not clear; however, lack of response and/or poor duration of antibodies to OPV at protective levels have already been described [56] and confirmed in the Italian population [57]. Moreover, several different factors, some of which are still unknown, may probably influence the immune response to the polio vaccine and the duration of anti-polio antibodies. This was shown by the relatively high rate of seronegative US military recruits observed in the 1970s [58] as compared with a sample of Italian subjects analyzed in the same period [59], who presented markedly lower rates of seronegativity, even though they were ranging in age from 6 months to 88 years. The response to the IPV booster was excellent and inversely associated with the baseline antibody levels (Table 3), as generally observed with other vaccines and with the OPV [56], but never, to the best of our knowledge, in the context of the OPV/IPV prime/booster. Despite the fact that the sequence IPV/OPV has been more extensively used, mainly for preventing vaccine-associated paralytic polio [60], the model of prime-boosting OPV-IPV has also largely been used and has been studied at infancy [61,62,63,64] with either a simultaneous OPV-IPV administration or administration of OPV-IPV delivered separately in a short time interval. However, to the best of our knowledge, no study has been conducted on the serum immunogenicity of the IPV booster administered to adults primed at infancy by OPV. In the current experience, this model has proven able to provide good results, considering that all group 1 subjects seroconverted (at least fourfold titer increase), at 1 month, to both serotypes, and at 9 months, to type 3, and all subjects but one seroconverted to type 1. Therefore, the IPV booster introduced by the Italian military health authorities in the compulsory vaccination schedule for the military in 1998 has shown to be capable of efficiently boosting the immune response towards the two types of polioviruses tested, without adverse effects. In fact, despite the continuous decline of wild polioviruses in circulation globally, maintaining a high level of immunity towards the three types of polioviruses is pivotal for the military. This can be considered as such not only in order to avoid possible reintroduction of the wild viruses in Italy as a consequence of infections taken during international missions abroad but also to prevent possible vulnerabilities, which may be exploited in case of malevolent use of polioviruses as biological weapons [57]. The lack of interference by the other concomitant vaccines may be inferred by the flat serum antibody titers observed in the seven subjects who had not received a recent polio booster. To the best of our knowledge, the post-vaccine serum antibody half-life has never been calculated; nevertheless, the long, even lifelong, persistence of anti-polio antibodies is well known [65], and thus, the relatively low half-lives calculated here are probably a consequence of the low number of explored subjects.

The model of influenza vaccination is particularly intriguing, considering that the immunization is repeated yearly due to the annual drifts; thus, the immune response and effectiveness are markedly influenced by the immune history of subjects [66]. In the current study, the immune response to the vaccine was good, considering that nearly 70% of subjects reached an antibody titer ≥ 1:40 towards all three H1, H3, and B antigens, which is generally considered putatively protective, even though the association between antibody titer and protection should only be considered as a guide [67] and not as absolute. In fact, it may depend on many causes, including the history of previous immunizations, which may negatively interfere with antibody production in cases of tight similarity between the viral strains circulating in two subsequent influenza seasons [66]. However, to underline the complexity of the influenza vaccination model, in cases of tight similarity between the viral strains circulating in subsequent influenza seasons, even an increase in baseline antibody levels has been described for a sort of cohort effect [68]. Additionally, even a mismatch between vaccines and circulating viral strains may be responsible for reduced immune response [66]. Conversely, the presence of the MF59 adjuvant provided a substantial contribution to the good antibody response [69]. Nevertheless, no case of ILI has been described in vaccinated individuals, despite the circulation of influenza in Lebanon in 2014 (mainly the H3N2 strain) [70]. It must be underlined that the influenza vaccine already showed good effectiveness in the military in 1944, inducing a 70% ILI reduction [25]. Moreover, no mismatch between vaccines and circulating viral strains was noticed in 2014. An inverse association between baseline antibody values and the level of immune response was confirmed, as already observed in patients with rheumatoid arthritis [68]. No interference by concomitant vaccines was observed, as documented by the results of the logistic regression.

The lack of association between HLA antigens and the immune response to the analyzed viral vaccines may perhaps be linked to the relatively low number of subjects explored, which prevented the possibility to study homogeneous subgroups consisting of enough subjects.

## 5. Conclusions

In conclusion, the current study confirms the immunogenicity of MMR, varicella, polio, HAV, and influenza vaccines in healthy adults, with an apparent lack of reciprocal interference, especially for polio, HAV, and influenza, and demonstrates that the inverse association between baseline antibody values and the level of immune response observed for bacterial antigens such as meningococcal [27], pneumococcal [71] and tetanus/diphtheria [28] is also detected for inactivated and live viral vaccines. This confirms the original observation of regulatory activity on the immune response by antibodies [72], which has been shown recently for the yellow fever vaccine [73] but has never been systematically explored. The Italian policy of vaccination in the military, irrespective of baseline antibody levels, should probably be reconsidered for MMR in light of the high prevalence of putatively protected individuals at baseline, which will probably be maintained and further increased in the future with the arrival of cohorts vaccinated at infancy.

## Figures and Tables

**Table 1:** Number and percentage of individuals receiving the indicated vaccines.

Vaccine	Trade Name	Adjuvant	Group 1 Subjects N (%)	Group 2 Subjects N (%)
Tetanus/diphtheria	DifTetAll	Alum	35 (32)	18 (25)
Inactivated polio	Imovax	-	26 (24)	26 (36)
Measles/mumps/rubella	Priorix	-	49 (45)	1 (1.4)
Chickenpox	Varivax	-	8 (7.4)	0
Hepatitis A	Epaxal	Virosomes	56 (52)	6 (8.3)
Hepatitis B	Engerix	Alum	0	3 (4.2)
Tetravalent Men Ps	Mencevax	-	103 (95)	65 (90)
Influenza	Fluad	MF59	0	72 (100)
Oral typhoid	Vivotif	-	0	51 (71)

-: Lack of adjuvant.

**Table 2:** Immune response to hepatitis A virus (HAV), measles, mumps, rubella, varicella, polio, and influenza vaccines.

Vaccine	N	N (%) Seropositive (Protected)			Putative Protective Level
		T0	T1	T2	
HAV	56	8 (14)	55 (98)	54 (96)	≥0.01 IU/mL
Measles	49	40 (81.6)	47 (96)	47 (96)	≥0.35 IU/mL (0.15–0.35 IU/mL equivocal)
Mumps	49	40 (81.6)	48 (98)	48 (98)	≥1:500 (1:230–1:500 equivocal)
Rubella	49	36 (73.5)	48 (98)	48 (98)	≥7 IU/mL (4–7 IU/mL equivocal)
Varicella	8	6 (75)	8 (100)	8 (100)	≥5 IU/mL
Polio 1	52	52 (100)	21 (100)	46 (100)	≥1:8
Polio 3	52	48 (92)	21 (100)	46 (100)	≥1:8
Influenza H1	72	44 (61)	ND	68 (94)	Seroprotection rate ≥ 70%
Influenza H3	72	28 (39)	ND	55 (76)	Seroprotection rate ≥ 70%
Influenza B	72	38 (53)	ND	59 (82)	Seroprotection rate ≥ 70%

ND: Not done.

**Table 3:** Demographic and immunization characteristics of subjects immunized with HAV and measles/mumps/rubella (MMR) vaccines.

	HAV		Measles		Mumps		Rubella	
	Responders	Non-Responders	Responders	Non-Responders	Responders	Non-Responders	Responders	Non-Responders
Military group	1st	1st	1st	1st	1st	1st	1st	1st
N (%)	46 (82)	10 (18)	5 (10)	44 (90)	7 (14)	42 (86)	12 (24)	37 (76)
Male sex N (%)	38 (83)	9 (90)	5 (100)	41 (93)	7 (100)	39 (93)	11 (92)	35 (95)
Mean age	20.60	20.70	19.60	21.86	21.57	21.64	21.25	21.76
Meningococcal N (%)	45 (98)	10 (100)	4 (80)	42 (95)	7 (100)	39 (93)	11 (92)	35 (95)
HAV N (%)	46 (100)	10 (100)	NA	NA	NA	NA	NA	NA
MMR N (%)	NA	NA	5 (100)	44 (100)	7 (100)	42 (100)	12 (100)	37 (100)
Varicella	NA	NA	1	6 (14)	3 (43)	4 (10)	2 (17)	5 (13.5)
Polio N (%)	29 (63)	7 (70)	1	14 (32)	2 (29)	13 (31)	5 (42)	10 (27)
Td N (%)	16 (35)	2 (20)	0	16 (36)	3 (43)	13 (31)	5 (42)	11 (30)
Mean N concurrent vaccinations	2.90	2.95	2.20	2.77	3.14	2.64	2.92	2.65
GMCs (IU/mL) or GMTs T0	0.00015 *	0.015	0.12 °	2.66	1:125 °	1:2930	1.29 °	58.77
T2/T0	602.25 ^	1.73	10.7 ^	0.98	11.11 ^	1.24	67.11 ^	1.23

* *p* = 0.0345 by logistic regression; ° *p* < 0.0001 by Student’s *t*-test on ln responders vs non-responders; ^ *p* < 0.00001 by Student’s *t*-test on ln responders vs non-responders; NA: not available.

**Table 4:** Demographic and immunization characteristics of subjects immunized with Polio and Influenza vaccines.

	Polio				Influenza					
	Type 1		Type 3		H1		H3		B	
					Responders	Non-Responders	Responders	Non-Responders	Responders	Non-Responders
Military group	1st	2nd	1st	2nd	2nd	2nd	2nd	2nd	2nd	2nd
N (%)	25	6	25	6	37 (51)	35 (49)	42 (58)	30 (42)	28 (39)	44 (61)
Male sex N (%)	18 (72)	5 (83)	18 (72)	5 (83)	35 (95)	34 (97)	39 (93)	30 (100)	26 (93)	43 (98)
Mean age	19.9	31.8	19.9	31.8	32	31	31.8	30.3	29.6	32.1
Typhoid N (%)	0	4	0	4	26 (70)	25 (71)	26 (62)	25 (83)	21 (75)	30 (68)
Meningococcal N (%)	24 (96)	4 (67)	24 (96)	4 (67)	31 (84)	34 (97)	37 (88)	28 (93)	26 (93)	39 (89)
HAV N (%)	20 (80)	0	20 (80)	0	5 (13.5)	1 (2.9)	3 (8.1)	3 (8.6)	5 (18)	1
HBV N (%)	0	0	0	0	3	0	3 (8.1)	0	2 (7)	1
MMR N (%)	4 (16)	0	4 (16)	0	0	1	0	1	0	1
Polio N (%)	25 (100)	6 (100)	25 (100)	6 (100)	6 (16)	6 (17)	7 (19)	5 (14)	4 (14)	8 (18)
Td N (%)	4 (16)	4 (67)	4 (16)	4 (67)	11 (30)	7 (20)	12 (32)	6 (17)	6 (21)	12 (27)
Flu N (%)	0	6 (100)	0	6 (100)	37 (100)	35 (100)	42 (100)	30 (100)	28 (100)	44 (100)
Mean N concurrent vaccinations	3.12	3.83	3.12	3.83	3.22	3.11	3.10	3.27	3.32	3.07
Mean N inactivated polio boosters	1	2	1	2	NA	NA	NA	NA	NA	NA
GMTs T0	36 *	90.5	23 *	128	20 #	112	11.6 °	40.9	18.1 ^	39.4
T2/T0	52 *	5	184 *	3.7	95 #	1.6	47.6 #	1.3	24 #	1.3

* p < 0.0001 by Student’s t-test on ln 1st vs 2nd group; # *p* < 0.0001, ° *p* = 0.0005, ^ *p* = 0.012 by Student’s t-test on ln responders vs non-responders; NA: not available.

**Table 5:** Influenza vaccine immunogenicity in 72 group-2 subjects according to the European Committee for Proprietary Medicinal Products (CPMP) parameters.

	H1/A/California/7/09		H3/A/Texas/50/12		B/Massachusetts/2/12	
	T0	T2	T0	T2	T0	T2
Seroprotection rate %	61	94	39	78	51	82
Seroconversion rate %	51		58		40	
Seroconversion factor	36		32		10	

**Table 6:** Logistic regression for influenza vaccine.

Responders at T2	H1N1 OR, 95% CI	H3N2 OR, 95% CI	B OR, 95% CI
Age	1.07, 0.93–1.23	1.03, 0.91–1.17	0.82, 0.70–0.95 ⁋
N of concurrent vaccinations	1.10, 0.53–2.26	0.83, 0.42–1.66	1.36, 0.67–2.75
H1 antibody T0	0.98, 0.97–0.99 *		
H3 antibody T0		0.97, 0.95–0.99 °	
B antibody T0			0.98, 0.97–1.00 ₸

* p = 0.0020; ° *p* = 0.0024; ⁋ *p* = 0.0074; ₸ *p* = 0.0261.
